# Editorial: Pediatric CNS tumors in low- and middle-income countries: expanding our understanding

**DOI:** 10.3389/fonc.2025.1548727

**Published:** 2025-01-27

**Authors:** Diana S. Osorio, Ibrahim Qaddoumi, Daniel C. Moreira

**Affiliations:** ^1^ Department of Pediatrics, The University of Texas MD Anderson Cancer Center, Houston, TX, United States; ^2^ Department of Global Pediatric Medicine, St. Jude Children’s Research Hospital, Memphis, TN, United States; ^3^ Department of Oncology, St. Jude Children’s Research Hospital, Memphis, TN, United States

**Keywords:** pediatric neuro-oncology, low- and middle-income countries, global oncology, capacity building, pediatric oncology

Pediatric central nervous system (CNS) tumors are a significant global health burden. These tumors are the second most common childhood cancer and the leading cause of cancer-related death in children (1). Each year, approximately 40,000 children worldwide are diagnosed, with substantial variations in incidence and outcomes between high-income and low- and middle-income countries (LMICs) (2). Many challenges exist, including late diagnosis, limited access to quality care, and lack of specialized treatment facilities (3, 4). Addressing this burden requires international collaboration, improved diagnostic and treatment capacity, all cemented on evidence-based approaches. Between May 2023 and October 2024 *Frontiers in Oncology* opened a Research Topic on *Pediatric CNS Tumors in Low- and Middle-Income Countries (LMIC): Expanding our Understanding*. Twenty-one manuscripts were published that provide insight into the challenges and advances in the care of children with CNS tumors across LMICs.

Pediatric oncologists Diaz-Coronado et al. leaders in their countries, provided an editorial summarizing the challenges contributing to the wide gap in survival outcomes in countries in Latin America. The lack of adequate infrastructure which may include an equipped neurosurgical center and intensive care unit beds, access to radiation, magnetic resonance imaging with timely reports, national treatment guidelines, lack of hospital beds and staff to care for children who may require high-dose chemotherapy or high-level inpatient care. Additional factors such as delays in diagnosis, limited access to medications, lack of a multidisciplinary team approach, higher rates of treatment abandonment, malnutrition, and lack of supportive care measures are common barriers.

Across LMICs, a significant limitation is the lack of pediatric cancer registries to provide real estimation of the burden of disease. However, there are two countries in the forefront of pediatric cancer registries in Latin America. The first and well-established Registro Oncopediátrico Hospitalario Argentino (ROHA) was started with Dr. Florencia Moreno with the support of the Kaleidos Foundation in 2000 in Buenos Aires, Argentina. ROHA unifies data from the various pediatric cancer centers across the country covering 93% of all national cases and since 2010 it grew to include children up to 19 years of age (5). The systematic collection of patient clinical data paired with diagnosis, pathology findings, institutional affiliation, care migration among other aspects has allowed for various analyses that help devise strategies relevant to the needs of their children and their families at the institutional, provincial and national levels. In Colombia, a Childhood Cancer Clinical Outcomes Surveillance System (VIGICANCER) was established in 2009 and currently obtains data from 27 pediatric oncology centers in ten Colombian cities. Ramirez et al.‘s manuscript was able to provide an estimate of the survival outcomes of children diagnosed with a pediatric brain tumor in Colombia. Data captured included mortality, relapse, treatment abandonment and occurrence of second neoplasms including rates of gross total resections. Their data revealed that survival estimates are lower than those of high-income countries.

The first national quantitative assessment of the pediatric neuro-oncology services and resources available in Mexico was undertaken. A total of 33 institutions participated, mostly from the public sector that care for much of the population. They reported that most institutions saw less than 10 new pediatric CNS tumors per year. Mexico reports a total of 850 newly diagnosed patients each year of which 300 deaths occur in children less than 19 years of age. UNICEF estimates there are 39 million children below the age of 18 years as of 2023 in Mexico. The incidence of pediatric brain tumors in the United States reported by CBTRUS in 2022 is approximately 6.3 cases per 100,000 children between 0-19 years of age. This would estimate there is a potential for 2,340 new diagnoses in Mexico between 0-18 years of age, suggesting there are a considerable number of children that are not being accounted for, diagnosed or seeking medical care in time. Of the resources mentioned, ICU bed and services were limited, pathology on average is based on basic histopathological testing, and nearly 20% of institutions did not have access to a neurosurgeon. This effort was published by Arce-Cabrera et al. to bring awareness of the current state of pediatric neuro-oncology resources in Mexico in the hopes to generate interest and amplify the critical components needed to be implemented for effective change in the care of children with CNS tumors.

Neuropathology has become increasingly sophisticated in the recent decade introducing molecular classifications in addition to histopathological analysis to arrive at an accurate diagnosis. In Latin America, it is very common to not have access to the basic immunohistochemical panels required for CNS tumors. Therefore, the likelihood being these rare tumors with great heterogeneity are being reviewed by a general pathologist in a center lacking expertise and equipment on histology alone. Some centers, like the National Children’s Hospital Dr. Carlos Sáenz Herrera in San Jose, Costa Rica, as described by Delgado given her additional training in pediatric neuropathology has been able to integrate a broad immunohistochemistry (IHC) panel but is still lacking in molecular studies, such as H3K27 or BRAF fusion studies among others. BRAFV600E is commonly available in Latin America since it is widely used in adult oncology, however, pediatric specific molecular studies are significantly lacking. There is awareness that molecular studies are not always going to help change your treatment management and traditional classifications through IHC are paramount, however, it will become imminent when molecular classification will be an essential component for diagnosis, treatment and prognosis in LMIC as well. Therefore, an excellent proposition by Dr. Nuñez to begin designating locoregional neuropathology centers of excellence and build capacity to review specimens for these highly specialized CNS tumors. It is unrealistic for pediatric cancer centers across LMICs to all become highly specialized in pediatric neuropathology.

Rajagopal et al. from Malaysia collected 50 medulloblastoma samples between 2003 and 2017 and were sent to Heidelberg, Germany for 850K Methylation. In their cohort of 48 patients, seven patients were treated as medulloblastoma, but methylation later revealed some discrepant results such as GBM, ATRT, Ewing sarcoma, MPNST, and pineoblastoma. They highlighted the importance of methylation in being able to subgroup their medulloblastoma samples and more accurately align their patient outcomes with the subgroups and in the future permit subgroup specific therapies, but they also recognize the high cost of this technology.

Amayiri et al. from The King Hussein Cancer Center wrote about their experience outsourcing molecular testing through their twinning program with SickKids Clinical Laboratory in Toronto. Of the 237 patients reviewed, 32 samples were sent for next generation sequencing based on the potential for clinical benefit. From this cohort they found 59% potentially actionable alterations, which included the use of targeted therapies and checkpoint inhibitors, three samples also revealing the suggestion of an underlying germline syndrome later confirmed with formal testing. The ideal would be for future evaluations that all samples be performed upfront rather than at progression.

Recognizing potentially actionable alterations are typically only beneficial if the local providers have access to the targeted medications. The King Hussein Cancer Center retrospectively reviewed their experience using BRAF/MEK inhibitors through a compassionate use program and provide outcomes after treating 20 patients (Laban et al.). Seventeen with 17 low-grade gliomas and three with high-grade glioma. Their experience with dabrafenib/trametinib was favorable with 47% of patients showing a favorable response to therapy vs. 11% for those who received chemotherapy and therefore a significantly longer median progression free survival (PFS) with dabrafenib/trametinib (20.1 months) compared to 7.4 months with chemotherapy. Unfortunately, access to these medications is a significant challenge since the compassionate use program has discontinued and the financial cost of these medications is prohibitive.

The article written by Gilani et al. described how they built neuropathology capacity at their center in Aga Khan University Hospital and Indus Children’s Cancer Hospital in Karachi, Pakistan (13). LMIC twinning with Sick Kids in Toronto provided training and mentorship to their pathologists. It also enabled infrastructure development by adopting and validating new immunohistochemical stains. Molecular diagnostics was undertaken at Sick Kids and their authors pointed out since precision therapies are still largely out of reach for most patients in LMIC, molecular biomarkers remained largely irrelevant for their capabilities as well as other LMIC. Nonetheless, they also found some unresolved cases where molecular techniques were indispensable for diagnosis. Thus, the development of affordable alternative molecular techniques will be important and concluding that select neuro-pathology reference labs in a particular country or region, will improve histopathologic diagnosis for LMIC.

Surgical challenges are vast across LMIC, Haizel-Cobbina et al. performed a large cross-sectional review of 312 patients treated across seven referral centers in Sub-Saharan Africa. She described a significant lack of neuronavigation, intraoperative imaging, and cortical mapping leaving neurosurgeons to depend on anatomical landmarks to perform their resections. Most patients also did not have access to post-operative imaging whether CT or by MRI. Overall, they found approximately one-third of patients indicated for surgery were unable to receive it. And most patients (74%-85%) for whom adjuvant therapy was recommended were unable to receive therapy. For those patients that managed to receive adjuvant care there was discoordination between the oncology and surgical teams leading to delays and missing the optimal window to administer adjuvant therapy.

Access to radiation therapy is also a challenge across LMIC with an insufficient number of radiotherapy machines as per the International Atomic Energy Agency (IAEA) and with technology that is not up to date. Additionally, there can be interruptions in therapy because machines frequently can be down in addition to the same issues with regards to expertise of personnel. Of note, there are some countries without radiation therapy at all. Therefore, LMIC-HIC partnerships and collaborations have proven crucial to address the gap in radiation therapy access. Hernandez et al. understand it is important to explore new, cost-effective radiation therapy technologies that would be more feasible for resource-limited settings. In their manuscript they were able to validate the use of an auto-planning tool for craniospinal radiation therapy planning. They utilized 3D-conformal CSI planning since 84% of resource-constrained clinics utilize this as opposed to more advanced techniques (ie: IMRT, VMAT). The efficiency of the tool has the promise to reduce contouring time and alleviate treatment delays which are known to impact survival outcomes, especially in LMICs.

Survival outcomes and other patient data for LMIC are significantly lacking in the literature. These data provide the pediatric oncology community with a better understanding of the circumstances experienced in the region and the strategies that need to be implemented for effective change. Additionally, it can provide a benchmark for which to measure the clinical impact of such treatment changes. In this series, although mostly retrospective analyses we are provided with clinical outcome data for patients with ependymoma, medulloblastoma, CNS germ cell tumors and optic pathway glioma (OPG) were written by their local pediatric oncologists.

Oigman et al. retrospectively reviewed 72 patients compiled over 20 years of data on pediatric patients with Ependymoma admitted to the National Cancer Institute of Rio de Janeiro, showing an OS for all patients of 67% at 5 years and 50% at 10 and 20 years. However, also demonstrating higher rates of recurrences and long-term quality of survival results inferior to HIC while also highlighting challenges in obtaining post-operative imaging and complete staging. In Peru, Perez-Roca et al. retrospectively reviewed 85 patients over the period of 19 years treated at the National Cancer Reference Center (INEN) in Lima with a 5-year OS for all patients of 55.89% and PFS 37.1%, finding challenges with only 31.76% of patients reported to have a gross total resection. Treatment abandonment was remarkably high in this cohort, as many as 23 patients (27%).

Ramirez-Melo et al. also retrospectively compiled data on 30 patients less than 18 years with newly diagnosed OPG from the Hospital Civil de Guadalajara, Mexico treated over 18 years. They were able to see that although they have the elements needed to provide favorable outcomes for their patients there are still barriers that lead to a poorer quality of survival such as high rates of surgical resections, post-surgical complications, and inability to assess functional outcomes such as vision in two-thirds of their patients. Additionally, they found a higher utilization of radiation therapy in up to 20% of patients contributing to the long-term burden of disease and a 10% rate of treatment abandonment.

Salceda-Rivera et al. compiled a large series of 284 patients treated in 21 pediatric oncology centers throughout Mexico between 1997 and 2017. This included infants and children up to 17 years of age treated with a variety of chemotherapy regimens, predominantly ICE, and up to 75% of patients received craniospinal radiation, including <3 years old. They reported an inferior survival in infants with desmoplastic nodular medulloblastoma of 58% 5-year OS, where in HICs OS is above 95% for non-metastatic non-p53 mutated desmoplastic nodular medulloblastoma utilizing radiation-avoidance chemotherapeutic regimens. However, this study now becomes a benchmark to help homogenize their national protocols and unify treatment strategies to improve survival outcomes, particularly for the most curable entities.

This series also included the Associate of Hematology/Oncology in Central America (AHOPCA) experience treating 48 patients over 20 years gathered mostly from Guatemala and Nicaragua and a few patients from El Salvador and the Dominican Republic. Giron et al. described a different reality where diagnosis is not able to be made with immunohistochemical stains rather relying on histology, serum tumor markers with clinical and imaging characteristics. They included all intracranial germ cell tumors reporting an OS of 68% for germinoma, 50.6% for NGGCT, and 85.7% for unclassified GCT.

Cappellano et al. shared their experience treating a series of complex, high-risk non-germinomatous germ cell tumors (NGGCT) at GRAACC, an experienced children’s cancer center in Sao Paulo, Brazil. A total of 15 patients with NGGCT were enrolled in their prospective trial that included all primary intracranial germ cell tumors (GCT). Most patients had pineal or suprasellar tumors and one bifocal. Three of these patients had metastatic disease, one with extra-neural metastasis to the lungs. Twelve patients with BHCG levels over 200 IU/L, seven with combined AFP levels >1000 ng/mL. They reported a 72% EFS and OS at 5 years for this notably high-risk population. This study also highlighted the risk of hyperhydration in a population of patients afflicted with diabetes insipidus as two toxic deaths occurred.

The therapies we administer are just as important as the supportive care measures we provide. Timely supportive care measures such as infusing antibiotics within 60 minutes of the onset of a fever is incredibly challenging for LMIC. Dassi et al. described the challenges they experienced with invasive fungal infections at GRAACC. An 11-year analysis of 818 children, of which 38 developed invasive fungal infections and concluded that careful evaluation of patient risk factors was the best mitigation strategy for prevention of this highly morbid and potentially fatal infection.

As mentioned, international collaborations between HICs and LMICs are also important to help manage rare, complex diagnoses. Daniel-Abdool et al. described their experience in Trinidad and Tobago caring for a child with WNT-Medulloblastoma and subsequently diagnosed with Turcot Syndrome. The diagnosis was made possible through a collaboration with Sick Kids in Toronto which allowed for the maximization of this child’s care.

Moreno et al. reviewed 266 medulloblastoma diagnosed in Brazil, Portugal and Argentina and they noted a higher incidence of hereditary WNT-activated medulloblastomas in the Latin-Iberian population in comparison to the North American and European populations. Interestingly, their Kaplan–Meier analysis revealed patients with WNT-activated medulloblastomas CTNNB1 wild-type had a worse outcome, with 71.4% in comparison to 100% of CTNNB1 mutant cases (p=0.031). Additionally, the WNT-medulloblastomas that are CTNNB1 wild-type cases can harbor APC germline mutations, suggesting that up to 27% of the Latin-Iberian cases of WNT-medulloblastoma may also have familial adenomatosis polyposis contrasting with 10% reported in North America and Europe. s

Robust multidisciplinary collaborations are essential to broaden our understanding of priority interventions and the implementation of successful programs. Over the past decade, we have witnessed a cohesive effort from pediatric oncologists and other pediatric subspecialists across Latin America to overcome the multiple barriers described. Baroni et al. from the Hospital Garrahan in Buenos Aires, Argentina described how they implemented a network and communication strategy through biweekly multidisciplinary meetings across their vast country that has enabled for an early referral system to improve the times to diagnosis and treatment strategies for children with CNS tumors. This effort spans the private and public sector and has proven to be beneficial and is to be implemented as a national health policy. In addition, a multidisciplinary collaboration in Pakistan sought to build pediatric neuro-oncology service delivery capacity by providing education programs, tumor boards, and strengthening of neuro-pathology review in collaboration with The Hospital for Sick Kids in Toronto through regularly scheduled multidisciplinary tumor boards (Mushtaq et al.). They concluded that the importance of establishing treatment protocols, fellowship programs, and regional tumor boards are sustainable opportunities to improve outcomes locally.

Children with CNS tumors in LMICs deserve quality care and should not be neglected as the field continues to advance and evolve. Our ability to cure these children should be constrained solely by our understanding of the disease’s biology, not by the availability of care. Ensuring equity in advanced treatments is crucial and probably the largest existing challenge in the field of pediatric neuro-oncology.
